# Electron spins from a molecular perspective: an interview with Song Gao

**DOI:** 10.1093/nsr/nwae327

**Published:** 2024-09-14

**Authors:** Shang-Da Jiang, He Zhu

**Affiliations:** NSR, Beijing, China; NSR, Beijing, China

## Abstract

Spin chemistry has emerged as an interdisciplinary field that focuses on electron spins in molecules and related applications in physics, chemistry, biology, materials science and information science. It will play a crucial role in technological innovations. The chemistry of spins is deeply related to the essence of chemical bonds, chemical catalysis, enzyme catalysis, optical and electromagnetic properties, quantum computation and quantum precision measurements. The related research is intersectional, cutting-edge and has a wide range of application prospects.

*National Science Review* (NSR) recently interviewed Prof. Song Gao to discuss spin chemistry. Prof. Gao is a renowned inorganic chemist and an academician of Chinese Academy of Sciences. He is the president of Sun Yat-sen University and the founder of the Spin-X Institute of South China University of Technology. He is recognized as a leader in coordination chemistry and molecular magnetism, and has strong academic influence. Since 2008, he has served on the International Advisory Committee of International Conference on Molecule-Based Magnets, and organized and chaired its 2010 conference. Prof. Gao's primary research topics include molecular nanomagnets; the relationship between geometric structures, electronic structures and magnetism of molecules; and the spin states and reactivity of molecules.

## THE ESSENCE OF SPIN CHEMISTRY RESEARCH

***NSR:*** What is the significance of electron spins in chemistry research?

***Gao:*** Spin is an intrinsic property of electrons and other fundamental particles at the quantum level. Since the birth of quantum mechanics approximately 100 years ago, the technological advancement of the world has paralleled with our growing knowledge of spins.

Research of traditional chemistry focuses on molecules’ geometric structures, steric effects, charge distribution and other aspects, and their effects on the physical and chemical properties of matter. Other traditional topics include product selectivity in reactions, reaction rates, chirality selectivity, etc. Electron spins in paramagnetic molecules provide a new degree of freedom in studies on the properties and reactivities of molecules. Appearing relatively rarely in present fundamental and applied studies, it is a challenging hotspot at the frontier of chemical research. Many important chemical reactions and catalytic processes involve open-shell complexes or organic radicals as intermediates. In processes such as catalytic synthesis and controlled radical polymerization, electronic spin states may determine the reaction speed and selectivity. Therefore, spin effects in chemical reactions can induce discoveries of new high-performance catalysts, synthesis methods and reaction paths.

***NSR:*** Physicists have studied spins extensively. What are the advantages of studying spins from a chemical or molecular point of view?

**Figure 1:**
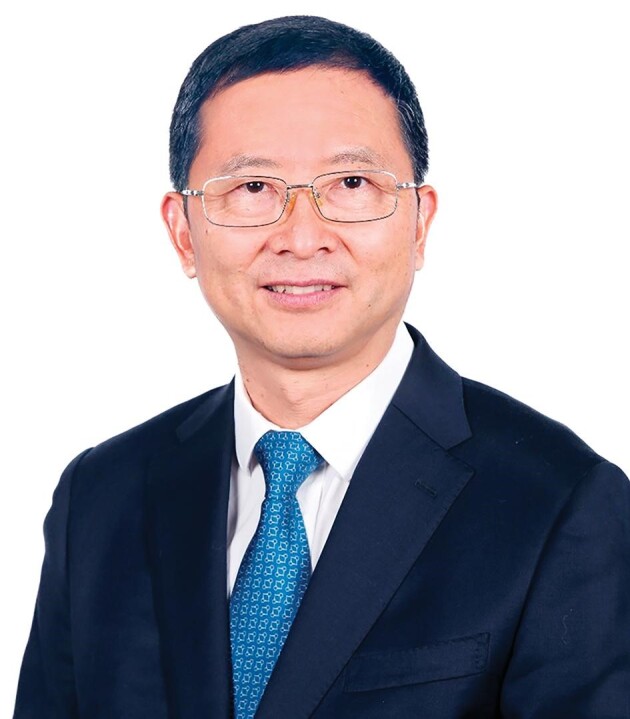
Professor Song Gao, a leading scientist in coordination chemistry and molecular magnetism (*Courtesy of Prof. Song Gao*).

***Gao:*** Spins have been a major focus in physical research, but physicists have mostly studied spins in relatively limited systems such as simple inorganic compounds, defects in crystals and

Spin chemistry emerged following collective progress in multiple fields.

—Song Gao

neutral atoms. As carriers of electron spins, molecular materials can be mass-produced and possess uniformity at an atomic level. Not only can this advantage help in our understanding of macroscopic magnetism from the atomic and molecular level, but it can also provide a solid foundation for the design and application of magnetic materials. In addition, molecules demonstrate outstanding designability such that their quantum behaviors can be modulated through chemical synthesis. Upcoming technologies in information sciences such as spintronics and quantum computation greatly rely on the research and development of spin materials. These spin materials, including molecule-based multiferroic materials, bipolar magnetic semiconductors and molecular qubits, provide important substrates for both classic and quantum information processing technologies.

In the past 20 years, interdisciplinary fusion has blurred the border between chemistry and biology. However, fusion between chemistry, physics and biology still has a long way to go. These interdisciplinary activities may allow researchers to solve long-standing problems such as the spin-related mechanisms in chemical reactions, catalysis or life. Molecular spin states are closely related to chemical processes and physiological functions including energy transfer and proton-coupled electron transfer in biological systems. Utilizing and manipulating molecular spin states help us to better understand phenomena related to spin and magnetism in biology and to monitor and modulate biological processes. These investigations may result in innovations in medical diagnosis and therapy with the potential for commercialization, leading to the prospect of future industries in the field of biomedicine.

***NSR:*** What specific research topics are included in spin chemistry?

***Gao:*** Spin chemistry emerged following collective progress in multiple fields including coordination chemistry, free-radical chemistry, chemical catalysis, biomedicine, semiconductor physics, quantum information and spintronics. It has grown into a vibrant cutting-edge multidisciplinary field. In general, spin chemistry includes, but is not limited to, the following five frontier areas: spin effects in chemical reactions, spin effects in molecular materials, new phases in molecular spin materials, molecular spins in diagnostic and therapeutic agents, and theoretical computation of molecular spins.

## MAJOR SCIENTIFIC QUESTIONS IN SPIN CHEMISTRY

***NSR:*** What scientific questions in spin chemistry are most important?

***Gao:*** In 2023, I organized an interdisciplinary frontier assessment project that was focused on spin chemistry in which we discussed emerging research fields concerning magnetic molecules. We held a series of eight sessions to discuss the interaction between molecular spins and electric fields, the interaction between molecular spins and light, intrinsic correlation between molecular spins and chirality, spin relaxation, new phases based on molecular spins, molecular spins in biomedicine, spin effects in chemical reactions and photoelectric radicals. More than 120 experts in chemistry, physics, biology, medicine and materials science attended the sessions. We spent nearly a year in literature review and discussions, and concluded that new science and technology in molecular spins have raised new questions on the fundamental properties and applications of magnetic molecules. For example, how can spin polarization be effectively achieved in magnetic molecules? What is the relationship between molecular spins and molecular chirality? How magnetoelectric coupling be achieved in a molecular system? These questions are crucial in multiple research fields and applications, and require conceptual breakthroughs at the intersection of many scientific disciplines.

***NSR:*** What new insights do we have regarding the relationship between spin and chirality in a molecular system?

***Gao:*** From a physical point of view, spin results from the breaking of time-reversal symmetry, while chirality results from the breaking of spatial inversion symmetry. In spin effects of chemical reactions or the manipulation of molecular spins, angular momentum can be transferred between different carriers such as a molecule or a photon, or between degrees of freedom such as spin and electron orbit. In practice, these phenomena point to new research opportunities in the systematic analysis of the relationship between electron spin, light polarization and molecular chirality. For example, the Faraday effect describes the coupling between spin state and light polarization, while circularly polarized emission and optical rotation result from the modulation of light by the chirality of materials’ structure. In recent years, Israeli scientist Ron Naaman discovered chirality-induced spin selectivity. It refers to the observation that non-polarized currents can become spin polarized through the interaction between the conducting electrons and the diamagnetic chiral molecules or structures. This effect directly connects microscopic spin states with structural chirality, while its origin can be traced to the transfer of angular momentum between electrons and chiral molecules.

From a physical point of view, spin results from the breaking of time-reversal symmetry, while chirality results from the breaking of spatial inversion symmetry.

—Song Gao

A fundamental understanding of chirality is still lacking. For example, we are still unable to determine the circular polarization properties of optical emission or absorption based on molecular chirality. This is because we do not have exact knowledge on how a chiral molecule responds to angular momentum. A deep understanding of the connection between chirality and electron spin and charge is also crucial in the investigation of chiral synthesis. In chemical reactions or catalytic processes that involve spin effects, spin polarization may induce chiral selectivity, and this may explain the origin of chirality in biological systems.

## UNIQUE FEATURES OF SPIN CHEMISTRY

***NSR:*** What is special about the research of spin chemistry?

***Gao:*** Spin chemistry now occupies a spot in science that interconnects with multiple disciplines. It is of significance to the nature of chemical bonds, chemical catalysis, enzyme catalysis, functional materials with opto-electromagnetic properties, quantum computation and quantum precision measurements. Research on spin chemistry features three characteristics:

*Interdisciplinary*: chemistry is a branch of science that creates and investigates new molecules. Spin-related phenomena, on the other hand, remain active areas of research in physics. In addition, many processes in life such as photosynthesis and bird navigation originate from molecular spin states, microstates and their dynamics. Magnetic molecules, as carriers of spins, play important roles in spintronics and quantum information. Therefore, the science and technology of spin chemistry thrive with the interactions between chemistry, physics, biology, materials science and information science.

*Innovative*: traditional chemistry has focused on electron charges in molecules and energy transfer in chemical reactions. Molecular spins carry and transfer angular momentum during chemical reactions and catalytic processes. So, in chemical reactions that involve spins, the transfer of angular momentum also needs attention. This is an aspect that should be emphasized in future research.

The science and technology of spin chemistry thrive with the interactions between chemistry, physics, biology, materials science and information science.

—Song Gao

*Applicable*: the scientific and technological outcomes from research on spin chemistry have great potential in promoting industrial advancements. For example, spin-induced chiral synthesis may reduce the difficulty of the synthesis of chiral drugs; understanding the mechanism of bird navigation may help to improve long-range navigation; molecular spin polarization may be used to develop core materials in MASER (microwave amplification by stimulated emission of radiation) to improve the detection sensitivity of extremely weak microwave signals; the DNP (dynamic nuclear polarization) technology may result in higher spatial resolutions in magnetic resonance imaging and bring about revolutionary improvement in medical diagnosis.

